# 2173: Investigation of sAC signaling reveals new therapeutic targets for cancer cell metabolism

**DOI:** 10.1017/cts.2017.25

**Published:** 2018-05-10

**Authors:** Jenny Wang, Jonathan Zippin

## Abstract

OBJECTIVES/SPECIFIC AIMS: The soluble adenylyl cyclase (sAC) is a noncanonical source of cAMP in mammalian cells. sAC is an ATP/bicarbonate ion sensor, whose activity responds to intracellular signals such as pH changes and metabolism. Unlike the more traditionally studied transmembrane adenylyl cyclase, sAC is not tethered to the cell membrane and instead is found in subcellular microdomains like the mitochondria and nucleus. In particular, sAC localization in the mitochondria has been implicated in oxidative phosphorylation and mitochondrial metabolism. Specific changes in sAC microdomain localization have diagnostic utility in a wide variety of cancers, namely melanoma. We have recently found that loss of sAC leads to tumorigenesis and a Warburg/cancer-like metabolic phenotype, consisting of an activated flux through glycolysis, increased lactate production, and dependence on glucose metabolism. In addition, computational analysis of the metabolomics profile of sAC null cells suggests an increased flux through serine synthetic pathways. We hypothesized that specific sAC microdomains are responsible for this cancer-like metabolic state. METHODS/STUDY POPULATION: We have established oncogenic SV40 large T antigen and HPV16-E6 expressing mouse embryonic fibroblasts lacking sAC expression (SV40 KO and E6 KO, respectively). Using these parental lines, we reintroduced sAC by targeting the protein to specific microdomains. sAC was either driven into the mitochondria (mito-sAC) or was driven into all possible microdomains (WT sAC). Single clones were generated and sAC expression was confirmed by Western analysis. Stable cell lines were evaluated for mitochondrial metabolism, glucose sensitivity, and serine sensitivity. RESULTS/ANTICIPATED RESULTS: We found that reintroduction of WT sAC into sAC null cells rescued sensitivity to glycolytic inhibition compared with control cells (*p*<0.01). The effect was not dependent on the method of immortalization as it was seen in both SV40 and E6 KO cell lines. sAC activity was not directly proportional to expression suggesting that additional regulatory pathways exist. Interestingly, targeted delivery of sAC to the mitochondria was not as effective in rescuing glucose sensitivity as untargeted delivery of sAC into all possible microdomains. Therefore, even though mitochondrial sAC is known to influence metabolism, our data suggests that the nonmitochondrial isoform is most important for cancer cell metabolism. Although metabolomics analysis suggested that serine synthetic pathways are activated in sAC null cells, there is no evidence to suggest that serine is required for sAC null cell growth. Neither inhibition of serine synthesis nor serine starvation differentially affected the growth of sAC null cells compared with WT sAC. DISCUSSION/SIGNIFICANCE OF IMPACT: These data suggest that the Warburg metabolic phenotype in sAC null cells is regulated by specific sAC microdomains. By targeting sAC to specific microdomains, we can further distinguish the role of sAC localization in cellular metabolism. Cancer cells have been shown to exhibit altered metabolic circuitry of pathways like glycolysis, which allow them to adapt to increased metabolic demands of cellular proliferation and waning environmental resources. Beyond helping us improve the use of sAC immunolocalization as a cancer diagnostic, a better understanding of sAC microdomains in transformed cells will help us understand how this signaling pathway is important in cancer. Pharmacologic manipulation of sAC signaling may represent a new cancer therapeutic strategy.

